# On the Impact of Substrate Uniform Mechanical Tension on the Graphene Electronic Structure

**DOI:** 10.3390/ma13204683

**Published:** 2020-10-21

**Authors:** Konstantin P. Katin, Mikhail M. Maslov, Konstantin S. Krylov, Vadim D. Mur

**Affiliations:** 1 National Research Nuclear University “MEPhI”, Kashirskoe Shosse 31, 115409 Moscow, Russia; KPKatin@mephi.ru (K.P.K.); krylov.const@gmail.com (K.S.K.); 2 Laboratory of Computational Design of Nanostructures, Nanodevices, and Nanotechnologies, Research Institute for the Development of Scientific and Educational Potential of Youth, Aviatorov str. 14/55, 119620 Moscow, Russia

**Keywords:** graphene, substrates, energy gap, Dirac velocity, mechanical deformation, critical charge, supercharged impurity, resonant scattering

## Abstract

Employing density functional theory calculations, we obtain the possibility of fine-tuning the bandgap in graphene deposited on the hexagonal boron nitride and graphitic carbon nitride substrates. We found that the graphene sheet located on these substrates possesses the semiconducting gap, and uniform biaxial mechanical deformation could provide its smooth fitting. Moreover, mechanical tension offers the ability to control the Dirac velocity in deposited graphene. We analyze the resonant scattering of charge carriers in states with zero total angular momentum using the effective two-dimensional radial Dirac equation. In particular, the dependence of the critical impurity charge on the uniform deformation of graphene on the boron nitride substrate is shown. It turned out that, under uniform stretching/compression, the critical charge decreases/increases monotonically. The elastic scattering phases of a hole by a supercritical impurity are calculated. It is found that the model of a uniform charge distribution over the small radius sphere gives sharper resonance when compared to the case of the ball of the same radius. Overall, resonant scattering by the impurity with the nearly critical charge is similar to the scattering by the potential with a low-permeable barrier in nonrelativistic quantum theory.

## 1. Introduction

Since its experimental synthesis in 2004 [1], graphene has attracted considerable attention from researchers due to its promising electronic properties. In terms of electronic characteristics, graphene is a gapless semiconductor [2]. While a traditional semiconductor such as silicon or germanium has an energy (semiconductor) gap, then graphene has a zero gap. In other words, the bottom of the conduction band and the ceiling of the valence band converge at one point in graphene, which is called the Dirac point. Moreover, ideal free-standing graphene possesses a linear dispersion relation in the vicinity of the Dirac point [3]. Charge carriers in graphene behave like massless relativistic particles that are called Dirac fermions, which determines their extremely high mobility. For example, the mobility of charge carriers in graphene reaches extremely high values, up to ~200,000 cm^2^V^−1^s^−1^ [4]. In other semiconductors (such as silicon or germanium), it is fifty or more times lower. At the same time, graphene has a number of other unique qualities. For example, Young’s modulus of graphene, which characterizes the material’s resistance to mechanical deformation, is higher than the corresponding values of steel and tungsten [5,6], and its thermal conductivity is significantly higher than the conductivity of traditional conductors such as copper or silver [7]. Unfortunately, the absence of a finite bandgap makes it impossible to get rid of leakage currents. This means that the current through graphene can never be turned off completely. The latter is a severe barrier to the graphene use in logic devices, e.g., transistors. Therefore, for the practical applications of graphene in nanoelectronics, a considerable energy gap should be opened. Various methods are proposed for introducing the semiconductor gap in graphene. Among them are chemical functionalization [8,9,10], the formation of graphene nanoribbons [11,12], mechanical strain [13,14], and the use of suitable substrates. It should be noted that the most proposed substrate for creating a semiconducting gap in graphene is a boron nitride substrate [15,16,17,18,19], but it is not the only possible one. Thus, properly chosen substrates can significantly change the electronic band structure of graphene [20], and, along with the mechanical stretching, it can become an alternative approach for straightforward tuning the electronic properties of graphene and obtaining a required bandgap (see Review [21] for details). Moreover, there are approaches that allow one to generate asymmetric deformation or doping between layers and methods for its quantitative determination using a supported isotopically labeled bilayer graphene studied by in situ Raman spectroscopy [22]. The mechanically controlled bandgap was previously observed in other 2D materials [23,24,25,26]. However, the exceptional mechanical properties of graphene [27,28] provide outstanding efficiency of mechanical strain engineering in this material.

In addition, graphene with a gap in the electronic spectrum in the presence of a multiple-charge impurity is of particular interest. Such a system is similar to the relativistic Coulomb problem [29]. In the two-dimensional case, the total angular momentum ℏJ=ℏ(M+1/2), where ℏM is the orbital angular momentum is a good quantum number by virtue of the axial symmetry [30]. In the three-dimensional problem, the Dirac quantum number ϰ is conserved due to spherical symmetry [31].

Since graphene is an almost ideal planar system, the orbital angular momentum may be quantized fractionally [32,33]. In addition, if one takes into account the motion reversal invariance, then M is limited to half-integer values only [34]. Therefore, the possible values of the total angular momentum J=M+1/2 in graphene are either half-integers or integers with the zero included [35]. Therefore, the radial two-dimensional Dirac equation, in the case of the Coulomb impurity, is identical to the three-dimensional one only for the integer nonzero values J=−ϰ= ±1, ±2, ….

It has long been known that pure Coulomb potential is an excessive idealization in the three-dimensional problem, which (for the ground state ϰ=−1) loses its meaning for a nucleus charge $Z>\alpha^{-1}\approx 137$ [36]. Here, $\alpha =e^{2}/\hbar c$ is the Sommerfeld fine structure constant, which, in graphene, is replaced by the effective fine structure constant $\alpha_{D}=e^{2}/\hbar v_{D}$, where $v_{D}$ is the Dirac velocity. Since $\alpha_{D}∼1$ in graphene, regularization of the Coulomb potential is required already for values of the impurity charge $Z\equiv Z_{0}/\epsilon ≳1$, where we take dielectric properties of the environment into account by means of the effective dielectric constant ϵ. Here, $Z_{0}$ is the “bare” impurity charge. According to Reference [37], the finite impurity sizes $r_{0}$ should be taken into account, which can be achieved by modifying the Coulomb potential at small distances.

The particular value $Z_{s}≃137$ in the three-dimensional problem corresponds to the singular effective charge value $Z_{s}\alpha_{D}=\left|J\right|$ in graphene. In two dimensions, the lowest energy level of the states with the total angular momentum J, described by the equivalent of the Sommerfeld formula [38,39] $E=m^{*}v_{D}^{2}\sqrt{J^{2}-Z^{2}\alpha_{D}^{2}}$ ($m^{*}$ is the effective mass of the charge carriers), vanishes at $Z=Z_{s}$ and is imaginary for $Z>Z_{s}$. Thus, to eliminate this difficulty and make the effective radial Dirac Hamiltonian self-adjoint, it is necessary to regularize the Coulomb potential [37].

After the regularization, a further increase in the charge results in the energy level below zero and, at the critical charge value [37] $Z=Z_{\mathrm{cr}}$, crossing the boundary of the lower continuum of the Dirac equation solutions, which, for the gapped graphene, is the ceiling of the valence band. The electron level disappears from the spectrum at $Z>Z_{\mathrm{cr}}$ [37], and the quasi-stationary state of the hole appears in the lower continuum instead. This picture is described in detail in Reference [39], and, in the semiclassical approximation, it is described in Reference [40]. In the latter case, the Dirac equation is reduced to the Schrödinger equation [41,42] in which the effective potential has a low-permeable barrier at E<0 [43].

Since the radial Dirac Hamiltonian is self-adjointed, one can interpret the solution of the Dirac equation with the energy $E<-m^{*}v_{D}^{2}$ as the state of a hole with positive energy $\bar{E}=-E>0$ scattering by a supercritical impurity. In this case, the complex energies $E_{p}=E_{0}-i\Gamma /2$ of quasidiscrete levels, where $E_{0}$ and Γ are their positions and widths, respectively, are given by the poles of the unitary partial elastic scattering matrix $S_{J}(\bar{E};r_{0})=\exp\{2i\delta_{J}(\bar{E};r_{0})\}$.

The Coulomb barrier is broad enough so that the quasistationary states have the small widths $\Gamma \ll E_{0}∼m^{*}v_{D}^{2}$, and Breit–Wigner resonances can arise in the scattering of holes, as in the nonrelativistic theory of scattering by a potential barrier with a low permeability [44]. More specifically, the elastic scattering phase $\delta_{J}(\bar{E};r_{0})$ sharply changes when the hole energy $\bar{E}$ is within the width Γ near the position $E_{0}$ of the quasidiscrete level [39], and the holes scattering partial cross-section comes close to the unitary limit. This can be detected in the current-voltage characteristics obtained experimentally by means of scanning tunneling microscopy [45].

**Figure 1:**
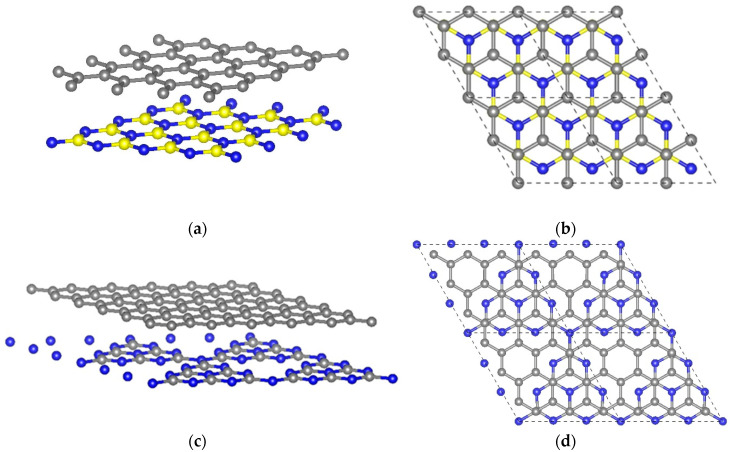
The atomic structures of hexagonal graphene supercells: graphene deposited on the hexagonal boron nitride (perspective view (**a**), top view (**b**)) and graphitic carbon nitride (perspective view (**c**), top view (**d**)). Gray, blue, and yellow balls represent C, N, and B atoms, respectively.

In the gapless case, one can legitimately call $Z_{s}$ the critical charge $Z_{c}$, as is often found in the literature [46,47], since the lower continuum states at $Z>Z_{s}$ are the scattering states of holes. The validity of the approach to describe the dynamics of charge carriers in the gapless graphene with Coulomb impurities using the radial Dirac equation was established in References [46,47], where theoretical calculations based on this assumption are in good agreement with the spectra of current-voltage characteristics dI/dV measured experimentally near the Dirac point. This is especially clearly manifested by the presence of a peak in the current-voltage characteristics measured near the center of a cluster containing five calcium dimers, which correspond to the scattering of a hole with J=1/2 by a supercritical impurity (see Figure 1E in Reference [46]). The emerging second peak is possibly related to the resonance in the state with J=1, i.e., with the half-integer value M=1/2 of the orbital angular momentum. There is not even a hint of its existence in theoretical calculations. Apparently, this is due to the fact that the scattering states considered in these calculations do not include ones with half-integer values of the orbital angular momentum, which are possible in two-dimensional problems (half-integer quantization of the orbital angular momentum is realized in circular quantum dots with an odd number of electrons [48,49]). In the gapless graphene, as opposed to the gapped one, the holes scattering by a charged impurity in the state with J=0 cannot lead to Breit–Wigner resonances and, therefore, to the peaks in the dI/dV spectra since the scattering phase in these states $\delta_{0}(E;r_{0})$ is smoothly dependent on energy [39]. Therefore, in the current work, we mainly focus on the state with J=0.

In the presented study, we analyze the electronic behavior of monolayer graphene on the hexagonal boron nitride (*h*BN) and graphitic carbon nitride (*g*C_3_N_4_) substrates under the mechanical biaxial strain in the frame of density functional theory, supplemented by the van der Waals dispersion corrections. It was obtained that the weak van der Waals interlayer interactions, alongside the uniform strain of the graphene sheet on the substrate, leads to the considerable bandgap. Moreover, the value of bandgap increases monotonically with an increasing stretch and vice versa. Note that, due to the remarkable mechanical properties, some substrates allow reversible stretching of graphene up to 30% [50,51]. Therefore, the gap can vary in a broad region. It should be noted that the *h*BN or *g*C_3_N_4_ substrate has a crucial role since free-standing graphene does not have an energy gap under uniform mechanical stretching. We discuss a method to smoothly change the electronic properties of graphene, which has a gap in the electronic spectrum by mechanical stretching or compression, which includes tuning the critical value $q_{\mathrm{cr}}=Z_{\mathrm{cr}}e^{2}/\hbar v_{D}$ of the effective charge $q=Z\alpha_{D}$ due to the change in the Dirac velocity $v_{D}$ as well as the effective mass $m^{*}$ of charge carriers. We suppose that the data obtained in the presented study can allow one to create an effective way of tuning the electronic characteristics of graphene for its further use in micro-electronic and nano-electronic as well as straintronics devices.

The rest of the presented article is organized as follows. In Section 2, we describe the ab initio technique that is used to analyze the electronic characteristics of graphene deposited on substrates and also explain the atomic structure of the samples. Section 3 gives a brief description of the Dirac equation properties and analytical results in the case of a supercritically charged Coulomb impurity. In Section 4, we discuss the theoretical results obtained. Finally, Section 5 provides concluding remarks on the presented study.

## 2. Materials and Methods 

To study the geometry and electronic structure properties of graphene on the different substrates, we used the ab initio approach, namely density functional theory (DFT) and its implementation in the program Quantum ESPRESSO ver. 6.5 [52,53]. We consider Perdew-Burke-Ernzerhof (GGA-PBE) functional for the description of exchange-correlation energy [54], and, for the electron-ion interaction, we use the projector-augmented-wave method (PAW) [55,56]. The kinetic energy cutoff of 120 Ry (1632 eV) was chosen. The weak van der Waals interactions between the non-covalently bound graphene sheet and substrate are taken into the D3 Grimme (DFT-D3) dispersion corrections [57]. DFT-D3 approach possess improved accuracy due to the use of environmentally dependent dispersion coefficients and the inclusion of a three-body component to the dispersion correction energy term. The interlayer distance between the “graphene-on-the-substrate” layers is equal to 20 Å, which provides sufficient space separation to avoid unphysical interactions. Thus, the lattice parameter optimization along the Z-axis becomes unnecessary. The geometry optimization of the unstressed graphene deposited on the substrate was carried out without symmetry constrains until the Hellman-Feynman forces acting on the atoms became smaller than 10^-6^ hartree/bohr. The parameters of the supercells were also optimized. However, we perform the structural relaxation of the samples strained uniformly along the lattice vectors in the XY-plane with fixed parameters of the supercell. For the *k*-point sampling of the Brillouin zone integrations, the 12 × 12 × 1 Monkhorst-Pack mesh grid [58] is used. For the non-self-consistent field calculations, the *k*-point grid size has been increased to 24 × 24 × 1. For the structural relaxation, the Methfessel-Paxton smearing [59] technique with the width of 0.02 eV was used, and, for the density of electronic states calculation, the Böchl tetrahedron method [60] was applied. The properties of the electronic structure were elucidated by analyzing the band structure of the samples and their density of electronic states.

First, we attempted to select such substrates that would facilitate the opening of an energy gap in graphene without imposing additional conditions, such as an external electric field or mechanical stresses. We examined about fifty different substrates, including the common SiO_2_ and SiC, as well as more complex systems such as Te_2_Mo, *h*BN/Ni(111), or C_3_B/C_3_N. At the level of theory used, we obtained that, only quasi-2D hexagonal boron nitride (*h*BN) and graphitic carbon nitride (*g*C_3_N_4_), opened the band gap in graphene. In all other cases, the “Dirac cone” on the electronic band structure conserved its original shape, and graphene remained a gapless semiconductor, or the Fermi level shifted to the conduction band. Thus, graphene began to exhibit a metallic nature.

Nevertheless, some previous studies revealed that the external electric field could effectively tune the energy gap in graphene on the SiO_2_ and SiC substrates [61,62,63] by the Fermi level shifting to the forbidden energy band. However, there are no additional conditions for bandgap opening in the case of *h*BN and *g*C_3_N_4_ substrates. Peculiarities of the interlayer interactions between the graphene and these substrates lead to the “pure gap” that is characteristic of the narrow-gap semiconductor.

We represent the graphene on the *h*BN and *g*C_3_N_4_ substrates by the hexagonal supercells. In the case of the *h*BN substrate, the supercell contains eight carbon atoms, and, in the case of the *g*C_3_N_4_ substrate, the graphene sheet inside the supercell is represented by 18 carbon atoms (see Figure 1). In both cases, periodical boundary conditions are applied. Such a size and shape of the supercells are suitable for modeling the uniform graphene sheet stretching on the substrate. We carried out the uniform stretching and compression of the graphene sheet by simultaneously increasing the lattice parameters of the supercell in the XY-plane and further optimizing the atomic positions in the supercell.

## 3. Theoretical Background 

The dynamics of massive charge carriers in the state with energy E and total angular momentum J in graphene in the presence of a multiply-charged impurity near the Dirac point is described in the continuous limit by the effective two-dimensional radial Dirac equation.

(1) $$\begin{array}{l} H_{D}\Psi_{\varepsilon ,J}(\rho )=\varepsilon \;\Psi_{\varepsilon ,J}(\rho ),\;\Psi_{\varepsilon ,J}=\left(\begin{array}{c} F(\rho )\\ G(\rho ) \end{array}\right),\\ H_{D}=\left(\begin{array}{cc} 1+V_{R}(\rho ) & \frac{J}{\rho }+\frac{d}{d\rho }\\ \frac{J}{\rho }-\frac{d}{d\rho } & -1+V_{R}(\rho ) \end{array}\right), \end{array}$$

See References [35,39,64]. Here:

(2) $$E=m^{*}v_{D}^{2}\varepsilon ,\;J=M+\frac{1}{2}=0,\;\pm \frac{1}{2},\;\pm 1,\;\ldots ,\;\left|x\right|=l_{D}\rho ,$$

where x=(x,y) is a 2D position vector, $l_{D}=\hbar /m^{*}v_{D}$ is the “Compton length” in graphene, and

(3) $$V_{R}(\rho )=-\frac{q}{R}(\begin{array}{cc} R/\rho , & \rho \;⩾\;R,\\ f(\rho /R), & \rho \;⩽\;R \end{array}$$

is modified at small distances (ρ ⩽ R≪1) of the Coulomb attraction potential [37].

(4) $$V_{C}(\rho )=-\frac{q}{\rho },\;q=Z\alpha_{D},\;\alpha_{D}=\frac{e^{2}}{\hbar v_{D}},\;q>0,$$

where $Z=Z_{0}/\epsilon$ is the effective charge, $Z_{0}$ is the “bare” impurity charge, $\epsilon =\left(1+\epsilon_{s}\right)/2$ is the effective dielectric constant, and $\epsilon_{s}$ is the dielectric constant of the substrate. Passing to the continuous limit is justified if

(5) $$a_{\mathrm{CC}}\ll r_{0}\ll l_{D},\;r_{0}=Rl_{D},$$

where $a_{\mathrm{CC}}$ is the distance between carbon atoms and $r_{0}$ is the cutoff radius.

The rectangular cutoff f(ρ/R)≡1 allows one to solve the system (1) analytically for any J≠0. For the case J=0, the analytical solution is possible with an arbitrary cutoff function [39]. Regularization of the Coulomb potential at small distances ensures the self-adjointness of the Dirac Hamiltonian $H_{D}$ and allows one to trace the motion of the level with the given E and J as the effective charge Z increases. In particular, at some critical value $Z_{\mathrm{cr}}$ [37], the electron level reaches the boundary of the lower continuum of the Dirac equation solutions, i.e., the upper boundary of the valence band.

The work [64] considers the effect of a supercharged Coulomb impurity $Z>Z_{\mathrm{cr}}$ on the system of Dirac charge carriers in graphene with a gap in the electronic spectrum. It particularly discusses the screening of the impurity charge by electrons produced together with holes from the Dirac sea, which is in complete analogy with the spontaneous production of the electron-positron pairs at $Z>Z_{\mathrm{cr}}$ in the relativistic Coulomb problem [43,65,66,67]. According to the review [67], the electron level collides with the positron level at $Z=Z_{\mathrm{cr}}$, and with a further increase in the charge $Z>Z_{\mathrm{cr}}$, it disappears from the spectrum [37], plunging into the lower continuum. The creation of a virtual electron-positron pair does not require the energy. The electron “lands” on the supercharged nucleus, becoming “superbound” [68], and the positron goes to infinity. Therefore, the issue “cannot be solved in the framework of the quantum mechanics of a single particle” [29].

However, due to the self-adjointness of the radial Dirac Hamiltonian, see Reference [39] and the mathematically rigorous paper [69], the one-particle approximation for the Dirac equation is valid not only for $Z\;⩽\;Z_{\mathrm{cr}}$, but also for $Z>Z_{\mathrm{cr}}$ (see References [35,70] for the case of short-range potential [71]). Qualitatively, this can be understood using the semiclassical approximation, when the system (1) near the boundary of the lower continuum of solutions to the Dirac equation is equivalent to the Schrödinger equation [41,42] with effective energy $E_{\mathrm{eff}}=\left({\bar{\varepsilon }}^{2}-1\right)/2$ and potential

(6) $$U_{\mathrm{eff}}(\rho ;\bar{\varepsilon };J)=-\frac{1}{2}V_{R}^{\;2}(\rho )-\bar{\varepsilon }V_{R}(\rho )+\frac{J^{2}}{2\rho^{2}},\;\bar{\varepsilon }=-\varepsilon >1,$$

(see Figure 1 in Reference [40]). This means that, at small distances, both the particle with energy ε and the anti-particle with energy $\bar{\varepsilon }=-\varepsilon$ is attracted to the center, in contrast to the Schwinger mechanism of the pair production [72], when a constant electric field breaks the virtual e^+^e^–^-loop. Therefore, the impurity charge screening mechanism specified in Reference [64], according to the scenario described in the review [67], cannot be realized [35]. Therefore, the one-particle approach for the effective radial Dirac equation (1) remains valid in the supercritical region $Z>Z_{\mathrm{cr}}$. This is necessary so that the local density of states in graphene can be directly extracted from the experiment [45].

All these conclusions remain valid for the gapless radial Dirac equation in the presence of a supercharged impurity $Z>Z_{c}=\left|J\right|\alpha_{D}$. Noteworthy is the work [46], which shows that theoretical calculations describing the electronic properties of gapless graphene agree with the experimental data on the spectra of current-voltage characteristics obtained by the scanning tunneling microscopy. Thus, for example, one can see a peak in the dI/dV spectra measured near the center of a cluster of five calcium dimers corresponding to the resonant scattering of a hole in the state with J=1/2, i.e., M=0 by a supercharged impurity (see Figure 1E in Reference [46]). However, there is no peak corresponding to the value J=0, i.e., M=−1/2 since the scattering phase, in this case, is a smooth function of the hole energy $\bar{\varepsilon }=-\varepsilon >0$ [39].

(7) $$\delta_{0}(\bar{\varepsilon };R)=Z\alpha_{D}[\ln(2\bar{\varepsilon }R)-c].$$

Here, the values c=1 and c=4/3 correspond to a uniform distribution of the impurity charge over the sphere and the ball of radius $r_{0}$, respectively.

The scattering phase for states with the total angular momentum J=0 is different for the graphene with a gap in the electronic spectrum. In this case, $\delta_{0}(\bar{\varepsilon };q;R)$ as a function of the hole energy $\bar{\varepsilon }$ abruptly changes when *q* is close to its critical value, which can correspond to Breit–Wigner resonances in holes scattering by the supercritical impurity (see Figure 7 in Reference [39]). Therefore, in the rest of this paper, we will focus on discussing states with J=0, i.e., half-integer orbital angular momentum M=−1/2. The electron level with such a value of *J* first descends to the boundary of the lower continuum, i.e., for the top of the valence band, see Figure 1 in Reference [39].

The critical charge $q_{\mathrm{cr}}(n)=Z_{\mathrm{cr}}(n)\alpha_{D}$ at which the *n*-th level with the total angular momentum J=0 reaches the boundary ε=−1, is found from the equation below.

(8) $$\arg\Gamma [2iq_{\mathrm{cr}}(n)]+q_{\mathrm{cr}}(n)\{f_{0}-\ln[2q_{\mathrm{cr}}(n)R]\}=\pi n,\;n=0,\;1,\;2,\;\ldots$$

Here, Γ(z) is the Euler gamma function, and the values $f_{0}=1$ and $f_{0}=2$ correspond to a uniform charge distribution over the sphere and the ball of radius $r_{0}=Rl_{D}$, respectively.

At $Z>Z_{\mathrm{cr}}$ the electron level disappears from the spectrum [37], and the system (1) at $\bar{\varepsilon }=-\varepsilon >1$ describes the scattering of a hole by a supercharged impurity. For the partial elastic scattering matrix, we have the following [35,39].

(9) $$S_{0}(k)=e^{2i\delta_{0}(k)}=\frac{F_{0}^{*}(k)}{F_{0}(k)},$$

where

(10) $$F_{0}(k)=-i[e^{\frac{\pi q}{2}-i\eta }\;a-e^{-\frac{\pi q}{2}+i\eta }\;b^{*}]$$

is the analog of the Jost function in the nonrelativistic scattering theory [44], and the following notation is used.

(11) $$\begin{array}{c} a=\frac{1+\bar{\varepsilon }-k}{\Gamma [1-iq(1-\bar{\varepsilon }/k)]},\;b=\frac{1+\bar{\varepsilon }+k}{\Gamma [1-iq(1+\bar{\varepsilon }/k)]},\\ e^{2i\eta }=e^{2iq[f_{0}-\ln(2kR)]}\frac{\Gamma (2iq)}{\Gamma (-2iq)},\;k=\sqrt{{\bar{\varepsilon }}^{2}-1}. \end{array}$$

The poles of the scattering matrix $F_{0}(k)=0$ lead to the equation for the spectrum of the complex energies

(12) $$\frac{1+\bar{\varepsilon }-k}{1+\bar{\varepsilon }+k}\cdot \frac{\Gamma [1+iq(1+\bar{\varepsilon }/k)]}{\Gamma [1-iq(1-\bar{\varepsilon }/k)]}=e^{-\pi q+2i\eta }.$$

The solutions give both the positions $\varepsilon_{0}$ and the widths γ of the quasi-discrete levels.

(13) $$\bar{\varepsilon }=\varepsilon_{0}-i\gamma /2,\;\varepsilon_{0}>1,\;\gamma >0.$$

Near the top of the valence band $Z-Z_{\mathrm{cr}}\ll 1$, due to the low permeability of the Coulomb barrier at ρ≫R in the effective potential (6), the width of the quasistationary state is small $\gamma \ll \varepsilon_{0}∼1$.

As in the nonrelativistic theory of scattering, see Chapter 13 in Reference [41], quasistationary states can manifest themselves as resonances in the hole scattering if its energy $\bar{\varepsilon }$ is within the region of a sharp change in the scattering phase. In this case, if the background phase is absent $\delta_{\mathrm{bg}}=0$, the partial cross-section is described by the Breit–Wigner formula.

(14) $$\sigma_{0}(\bar{\varepsilon })=\sin^{2}\delta_{0}=\frac{{\left(\gamma^{*}/2\right)}^{2}}{{\left(\bar{\varepsilon }-\varepsilon_{0}^{*}\right)}^{2}+{\left(\gamma^{*}/2\right)}^{2}},$$

The position of the resonance $\varepsilon_{0}^{*}$ follows from the equality $\delta_{0}(\varepsilon_{0}^{*};q;R)=\pi /2$ when the scattering phase changes abruptly from 0 to π over the width $\gamma^{*}$. At the same time, the width $\gamma^{*}$ is determined from Equation (12) for such an effective charge q close to $q_{\mathrm{cr}}$, which corresponds to the value of $\varepsilon_{0}^{*}$. This leads to the appearance of peaks in the current-voltage characteristics measured near the center of the supercharged impurity in graphene. However, when the background phase $\delta_{\mathrm{bg}}\neq 0$, and then near the resonance $\bar{\varepsilon }\approx \varepsilon_{0}^{*}$, the total phase $\delta_{0}(\bar{\varepsilon };q;R)$ increases sharply from $\delta_{\mathrm{bg}}$ to $\delta_{\mathrm{bg}}+\pi$, which leads to a change in the shape of the resonance, and the Breit–Wigner formula is replaced by a more general one (see Reference [44]).

In the review [67], resonant scattering by supercritical nuclei was associated with the production of e^+^e^–^-pairs, and the width γ was considered as the probability of such a process. This point of view is shared by the authors of Reference [73]. However, due to the self-adjointness of the radial Dirac Hamiltonian, the scattering phase $\delta_{0}(k;q;R)$ is real, and the partial elastic scattering matrix $S_{0}(k)=\exp[2i\delta_{0}(k)]$ is unitary. Therefore, there are no inelastic processes, including spontaneous pair production. This statement applies to any value of the total angular momentum J [39]. In the gapless case, this fact was confirmed experimentally [46] for the states with J=1/2.

## 4. Results and Discussion

First of all, we determined the unstrained reference configuration of the graphene sheet on the *h*BN and *g*C_3_N_4_ substrates by optimizing the supercell parameters as well as atomic positions inside the supercell. For considered samples (see Figure 1), we chose the types of packing, that is, the mutual arrangement of graphene atoms and the substrate, which correspond to the lowest total energy and, therefore, are more thermodynamically stable [74,75]. The structure of graphene on the *h*BN is characterized as follows: one carbon atom is located directly above the boron atom, and the other carbon atom is located above the center of the *h*BN hexagon (see Figure 1). In the most energetically favorable graphene/*g*C_3_N_4_ system, graphene carbon atoms are located directly above the carbon atoms of the substrate and above the center of the C-N hexagon (see Figure 1). It should be noted that graphene conserves its plane structure, and there is no covalent bonding between graphene and substrate. In the case of the *h*BN substrate, the intercarbon bond length in the unstrained graphene is equal to 1.435 Å, and the distance between the graphene sheet and substrate is equal to 3.429 Å (experimental value is equal to 3.3 Å [76]). In the case of the *g*C_3_N_4_ substrate, the intercarbon bond length in the unstrained graphene is equal to 1.407 Å, and the distance between the graphene sheet and substrate is equal to 3.386 Å (experimental value is equal to 3.325 Å [77]). It should be noted that the lattice parameters of *g*C_3_N_4_ are about three times larger than the corresponding values for graphene. This means that for 1 × 1 supercell of the substrate corresponds to 3×3 supercells of graphene. Taking this into account, the mismatch of the lattice parameters of the supercells for graphene and *g*C_3_N_4_ substrate is about 3.6%. The lattice constants for graphene and hexagonal boron nitride correspond much better to each other. Their discrepancy is only 1.8%. In addition, the *g*C_3_N_4_ substrate contains large holes. It is “atomically inhomogeneous” in contrast to the *h*BN substrate, which leads to slight distortions of the graphene sheet. However, the intercarbon bond length in the unstrained graphene is in good agreement with the previously obtained experimental data [78,79]. All subsequent deformations and strains are defined with respect to this equilibrium honeycomb structure.

**Figure 2:**
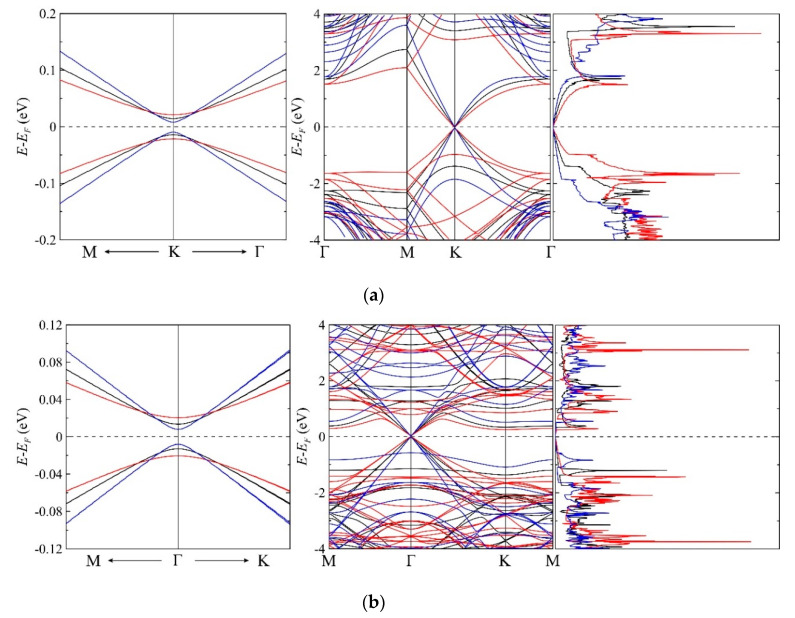
Band structure near the Dirac point (left), band structure (center), and density of electronic states (right) of the graphene deposited on the *h*BN (**a**) and *g*C_3_N_4_ (**b**) substrates. Blackline corresponds to the unstrained graphene, the blue line corresponds to the 10% compression, and the red line corresponds to the 10% stretching of the graphene sheet on the substrate. Γ, K, and M are the standard notations for the high-symmetry characteristic points in the Brillouin zone, where Γ corresponds to the center of the Brillouin zone. The Fermi level is assigned at zero.

At the next step, we analyze the electronic characteristics, namely band structure and density of electronic states, of the equilibrium honeycomb structure of graphene sheet on the *h*BN and *g*C_3_N_4_ substrates (see Figure 1). In both cases, we observe the energy gap. The presence of the *h*BN substrate leads to the semiconducting gap in graphene that is equal to 22.9 meV, and the presence of the *g*C_3_N_4_ substrate opens the semiconducting gap that is equal to 22.2 meV. Therefore, *h*BN and *g*C_3_N_4_ substrates contribute to a zero-gap semiconductor to a narrow-gap semiconductor transition. In the case of the hexagonal boron nitride substrate, it was previously shown that the mechanism of the energy gap appearing is concerned with the charge redistribution in the graphene layer and charge transfer between graphene and boron nitride layers by modifying the on-site energy difference of carbon *p* orbitals at two different sublattices [80]. It should be noted that, contrary to the free-standing graphene or the graphene on the *h*BN substrate, the band structure of the graphene on the *g*C_3_N_4_ substrate possesses a characteristic feature: the Dirac cone at the K point in the unit cell of graphene moves to the Γ point. The corresponding data are shown in Figure 2.

**Figure 3:**
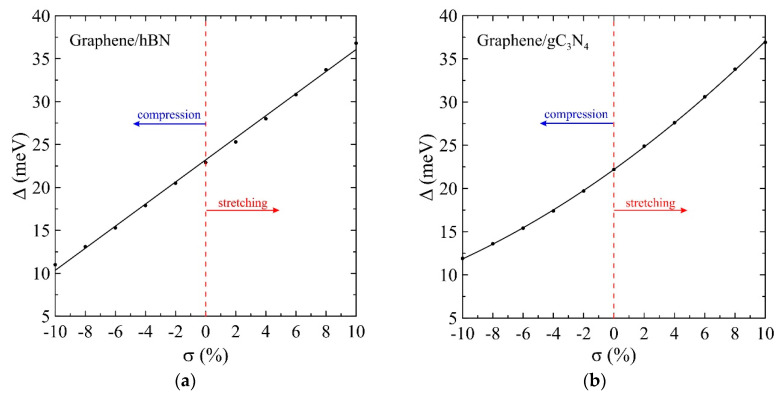
Semiconducting energy gap dependence on the uniform biaxial strain of the graphene deposited on the *h*BN (**a**) and *g*C_3_N_4_ (**b**) substrates. Circles are the results of the calculation, and the solid lines are the least-squares linear (**a**) and quadratic (**b**) fits.

Our subsequent studies are focused on the effects of mechanical stretching and compression on the behavior of the energy gap in graphene on the *h*BN and *g*C_3_N_4_ substrates. We analyze the impact of uniform biaxial compression and stretching to the electronic characteristics of graphene. These properties differ significantly from those of free graphene. Earlier, it was obtained that the uniaxial (zigzag or armchair) stretching and shearing as well as inhomogeneous deformation opens the energy gap in free-standing graphene [81,82,83]. On the contrary, isotropic biaxial stretching up to 20% left the graphene gapless [40]. This is due to the fact that, in the case of uniform stretching, the initial symmetry of the graphene lattice is retained, and, therefore, the band gap does not appear. On the contrary, when uniaxial deformation is applied, the graphene lattice symmetry decreases. Such deformations affect the irreducible part of the first Brillouin zone. It varies from its original triangular to the polygonal shape. Thus, the tops of the Dirac cones are no longer located at the high-symmetry points. They move along the Brillouin zone, either for deformations in the armchair or zigzag direction, and the energy gap appears. This is also true in the presence of the other layer or substrate. This effect is observed for the case of twisted bilayer graphene [84]. From a geometrical point of view, the symmetry breaking caused by the presence of the substrate also leads to the appearance of a gap. This effect also conserves during the uniform deformation of the graphene-substrate sample. Therefore, the presence of *h*BN or *g*C_3_N_4_ substrate makes it possible to tune the bandgap in graphene. In this case, stretching leads to an increase of the gap value, and compression leads to its decrease. For example, uniform stretching of the graphene sheet on the *h*BN substrate results in the gap of 36.8 meV, and, on the *g*C_3_N_4_ substrate, results in the gap of 36.9 meV (see Figure 3). These values are higher than thermal energy at room temperature (*k*_B_*T* ~26 meV), which indicates the possibility of maintaining the energy gap at normal conditions. Note that, in real straintronic applications, the limits of strain transfer between graphene and substrate should be considered since biaxial deformation is not always completely transferred from the substrate to graphene [85]. We plot the band structures and densities of electronic states of unstrained graphene, graphene stretched by 10%, and graphene compressed by 10% on the *h*BN and *g*C_3_N_4_ substrates (see Figure 2). Note that the bandgap of graphene on *g*C_3_N_4_ grows faster than the bandgap of graphene on *h*BN under stretching (see Figure 3).

**Figure 4:**
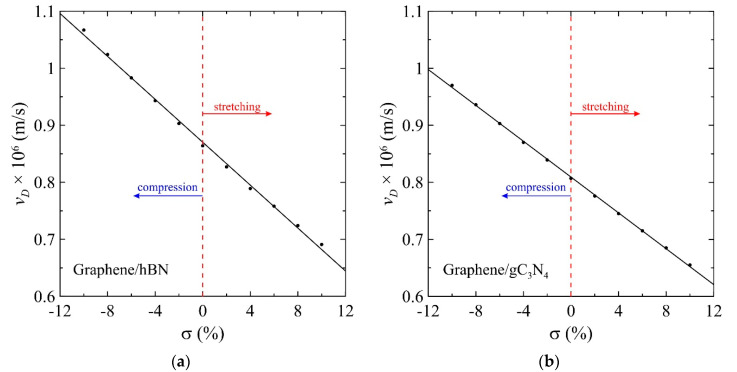
Dirac velocity dependence on the uniform biaxial strain of the graphene deposited on the *h*BN (**a**) and *g*C_3_N_4_ (**b**) substrates. Circles are the results of the calculation and the solid lines are the least-squares linear fits.

Next, we try to estimate the behavior of the Dirac velocity of unstrained and uniformly stretched/compressed graphene on the *h*BN and *g*C_3_N_4_ substrates. It is already known that, in a gapless free-standing graphene energy dispersion relation has a linear form, i.e., $E\left(k\right)=\hbar v_{F}k$, where $v_{F}$ is Fermi velocity, *k* is the modulus of the wave vector in two-dimensional space measured from the Dirac points, and ℏ is the reduced Planck’s constant. Thus, it can be said that electrons in pristine graphene have zero effective mass [86]. However, if graphene has a semiconducting gap, as in our case, when it is located on the *h*BN or *g*C_3_N_4_ substrate, the energy dispersion relation can no longer be considered linear near the Dirac points, and the electrons have a nonzero effective mass. In this case, we can approximate the bottom of the conduction band and the top of the valence band $\left(k=k_{0}\right)$ by the parabolic functions and estimate the effective mass of particles from the expression $m^{*}=\hbar^{2}{{\left(\frac{\partial^{2}E\left(k\right)}{\partial k^{2}}\right)}^{-1}|}_{k=k_{0}},$ where p=ℏk is the momentum of the electron. On the other hand, taking into account that the effective mass of charge carriers at the Dirac point given by equation $m^{*}\approx \frac{\Delta }{2v_{D}^{2}}$ [18], where Δ is the value of the energy gap, we can estimate the Dirac velocity $v_{D}$. Here, we use the notation for the Dirac velocity to avoid the confusion with the Fermi velocity that is traditionally used for the gapless free-standing graphene. Calculated results for the Dirac velocity for graphene on the *h*BN and *g*C_3_N_4_ substrate under uniform compression and stretching up to 10% are presented in Figure 4. The obtained data for the Dirac velocity depending on mechanical tension σ can be fitted with the following linear equation:

(15) $$v_{D}\left(\sigma \right)\left[\mathrm{Mm}/s\right]=-0.0188\sigma +0.8703$$

for the graphene on the *h*BN substrate, using the following linear equation.

(16) $$v_{D}\left(\sigma \right)\left[\mathrm{Mm}/s\right]=-0.0157\sigma +0.8092$$

for the graphene on the *g*C_3_N_4_ substrate. Thus, uniform stretching can be an effective instrument for the precise tuning of the energy gap of graphene on the considered substrates as well as the Dirac velocity.

As can be seen from Figure 2, the band structure near the Dirac point in the corner of the Brillouin zone for graphene on the *h*BN substrate is not very different from the graphene on the *g*C_3_N_4_ substrate. However, the Dirac point in the latter case is located in the center of the zone. Therefore, the presented results for the electronic structure described by the two-dimensional radial Dirac equation are limited only to graphene on the *h*BN substrate.

**Figure 5:**
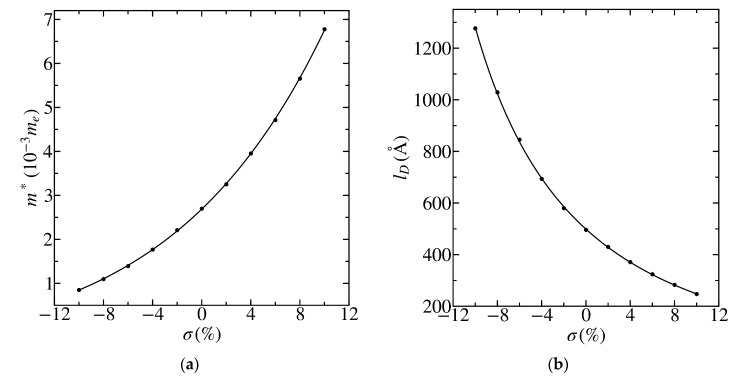
Dependence of the effective mass $m^{*}=\frac{\Delta }{2v_{D}^{2}}$ (**a**) and the “Compton length” in graphene $l_{D}\equiv \frac{\hbar }{m^{*}v_{D}}=2\hbar \frac{v_{D}}{\Delta }$ (**b**) on mechanical tension σ for graphene deposited on the *h*BN substrate. The circles correspond to the results calculated for Δ and $v_{D}$. The solid lines correspond to their least-squares quadratic fits (see Figure 3 and Figure 4).

Figure 3 and Figure 4 show that the gap Δ and Dirac velocity $v_{D}$ under stretching and compression in the range from σ=−10 % to σ=10 % change in opposite directions, which results in much larger regions of variation for the effective mass of charge carriers $m^{*}=\frac{\Delta }{2v_{D}^{\;2}}$ and the “Compton length” in graphene with a gap in the electronic spectrum $l_{D}\equiv \frac{\hbar }{m^{*}v_{D}}=2\hbar \frac{v_{D}}{\Delta }$ (see Figure 5). A continuous transition to the two-dimensional radial Dirac equation near the Dirac point is justified if $a_{\mathrm{CC}}\ll r_{0}$. The value $r_{0}=15\;Å$ that is used for calculations in this work, as well as in Reference [46] for the gapless graphene, is very acceptable here since the distance between carbon atoms $a_{\mathrm{CC}}∼1.5\;Å$ at σ=0 % in the considered cases. Stretching and compression by 10% changes these values by no more than 10% so that the dimensionless cutoff radius $R=r_{0}/l_{D}$ can vary over a wider region.

**Figure 6:**
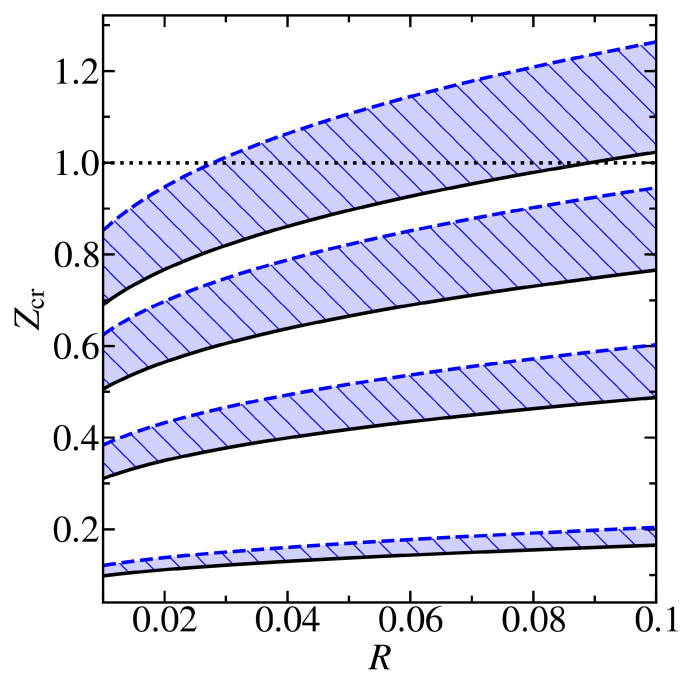
Dependence of the critical charge $Z_{\mathrm{cr}}(n)$ on the dimensionless cutoff radius $R=r_{0}/l_{D}$ with the cutoff function corresponding to the value $f_{0}=2$, i.e., to the uniform distribution of the impurity charge over the ball of radius $r_{0}$ for graphene on the *h*BN substrate. The shaded areas correspond to the first four levels n=0, 1, 2, 3 with the total angular momentum J=0. The solid black lines correspond to the absence of deformation σ=0 %. The dashed blue lines correspond to the compression σ=−10 %. The dotted line marks the closest integer value of the impurity charge Z=1.

Figure 6 shows the critical charge $Z_{\mathrm{cr}}(n)$, which is subject to Equation (8), as a function of the radius R for graphene deposited on the *h*BN substrate. Here, the cutoff function f(ρ/R) for the Coulomb potential (3) matches the uniform distribution of the impurity charge over the ball of radius $r_{0}$ and corresponds to the value $f_{0}=2$ in Equations (8) and (11). It is assumed that the effective dielectric constant ϵ=1. As one can see, the closest integer value of the impurity charge Z=1 is already supercritical for the first three levels n=0, 1, 2 with the total angular momentum J=0. For the level n=3, it can be both supercritical and subcritical, depending on the mechanical tension σ and the cutoff radius R.

**Figure 7:**
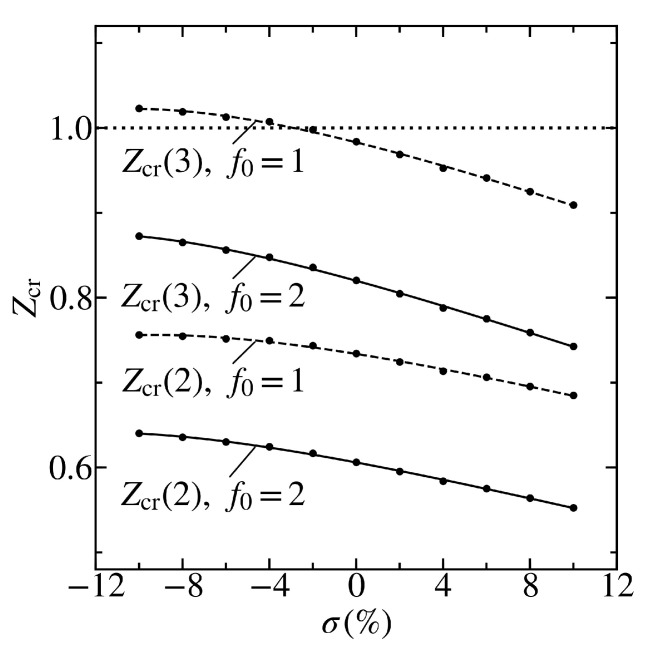
Dependence of the critical charge $Z_{\mathrm{cr}}(n)$ on the mechanical tension σ for graphene on the *h*BN substrate. The lines are shown for the levels n=2, 3 with the total angular momentum J=0, and for the cutoff function corresponding to the value $f_{0}=2$ (solid lines) and the rectangular cutoff with $f_{0}=1$ (dashed lines). The cutoff radius is equal to $r_{0}=15\;Å$. The circles correspond to the results calculated for Δ and $v_{D}$. The lines correspond to their least-squares quadratic fits (see Figure 3 and Figure 4). The dotted line marks the closest integer value of the impurity charge Z=1.

Figure 7 depicts the dependence of the critical charge $Z_{\mathrm{cr}}(n)$ on the uniform deformation σ of graphene on the *h*BN substrate at the cutoff radius $r_{0}=15\;Å$ for the levels n=2, 3 with J=0. It can be seen that the uniform stretching/compression results in a decrease/increase in the critical charge $Z_{\mathrm{cr}}$ both for the rectangular cutoff of the Coulomb potential (3) $f_{0}=1$, and for the cutoff function corresponding to the distribution of the impurity charge over the ball $f_{0}=2$. This statement also holds for the first two levels, except for the ground level when $f_{0}=1$ (see Table 1 and Figure 6). The impurity charge Z=1 is supercritical for $f_{0}=1$ and the level n=2 in the entire region of the considered deformations, and for the third excited level, when passing from σ=−10 % to σ=10 %, the value of Z=1 from subcritical becomes supercritical. At the same time, the charge Z=1 is supercritical in the entire range of σ for the cutoff function with $f_{0}=2$ and the level n=3.

**Figure 8:**
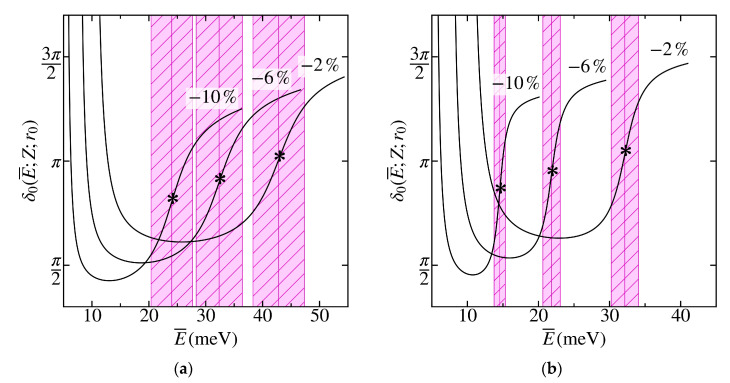
The scattering phase $\delta_{0}(\bar{E};Z;r_{0})$ as a function of the hole energy $\bar{E}$ in states with the total angular momentum J=0 for graphene on the *h*BN substrate. The impurity charge Z=1, the cutoff radius $r_{0}=15\;Å$, and the cutoff function corresponds to the uniform charge distribution over the sphere of the radius $r_{0}$ (**a**) and over the ball of the same radius (**b**). The asterisks mark positions of the resonances $E_{0}^{*}$ corresponding to the quasi-stationary states with n=2 (**a**) and n=3 (**b**), the shaded areas mark their widths $\Gamma^{*}$. The numbers indicate the compression values σ.

Figure 8 shows the phase $\delta_{0}(\bar{E};Z;r_{0})$ (see Equation (9)) of hole scattering by the impurity with the charge Z=1 in graphene on the *h*BN substrate for the states with the total angular momentum J=0 for those values of uniform deformation σ for which $Z-Z_{\mathrm{cr}}\ll 1$, i.e., when $\delta_{0}$ changes sharply in the region of the resonance $E_{0}^{*}$ over the width $\Gamma^{*}$ (see Equations (13) and (14)). Figure 8a corresponds to the resonant scattering of holes at energies close to the quasi-discrete level with n=2 in the case of the rectangular cutoff $f_{0}=1$ of the Coulomb potential (3), and Figure 8b corresponds to the resonant scattering of holes near the quasi-discrete level with n=3 and with the cutoff function matching the uniform distribution of the impurity charge Z over the ball $f_{0}=2$. These quasistationary states with complex energies (13) are described by Equation (12) and correspond to the poles of the partial elastic scattering matrix $S_{0}(k)$ (9).

It can be seen that the resonance $E_{0}^{*}$ shifts under the uniform compression toward lower values and becomes sharper, i.e., its width $\Gamma^{*}$ shrinks, which is due to a decrease in the supercriticality $Z-Z_{\mathrm{cr}}$ (see Figure 7). In addition, when replacing the cutoff corresponding to $f_{0}=2$ with the rectangular one $f_{0}=1$, the resonance becomes sharper if it does not disappear completely, i.e., if the quasidiscrete level does not become the discrete one with $\bar{E}=-E<m^{*}v_{D}^{2}$. In contrast to the behavior of the phase at resonance corresponding to the quasidiscrete level with n=0 (see Figure 7 in Reference [39]), where an abrupt change occurs from the value $\delta_{0}=0$ to $\delta_{0}=\pi$, in this case, the phase sharply changes from the value $\delta_{0}\approx \pi /2$ to the value $\delta_{0}\approx 3\pi /2$, which, apparently, is associated with the accumulation of the background phase $\delta_{\mathrm{bg}}$ when the level n rises. This means that the resonance should correspond not to the “pure” Breit–Wigner one (14), but to a more general case. In particular, it should correspond to a sharp minimum in the hole scattering cross-section $\sigma_{0}$ (see Reference [44] and Figure 13.3 therein for details).

## 5. Conclusions

In the presented study, we tried to answer the question of how the choice of the substrate and uniform mechanical stresses can affect the electronic properties in graphene, and, in particular, opens the energy gap and contributes to its fine-tuning. It was found that, when choosing the hexagonal boron nitride (*h*BN) and graphitic carbon nitride (*g*C_3_N_4_) substrates, the behavior of the electronic properties of deposited graphene is fundamentally different from the free-standing one. In this case, graphene has an energy gap, which makes it possible to classify it as a narrow-gap semiconductor, whereas the uniform stretching increases the value of the gap. These findings can help to overcome the main barrier to using graphene as an element of nano-electronic and straintronic devices. Moreover, uniform stretching affects the Dirac velocity in graphene on the substrate. Dirac velocity is linear with mechanical tension. It increases with uniform compression and decreases with uniform stretching. The Dirac point, near which the dispersion law is similar to the Einstein dependence of the particle energy on its momentum, corresponds, as usual, to the corner of the Brillouin zone for graphene deposited on the *h*BN substrate, while, for the *g*C_3_N_4_ substrate, it is located in the center of the zone. However, in both cases, the dynamics of charge carriers near the Dirac points is described by the effective two-dimensional radial Dirac equation for states with the total angular momentum J=M+1/2. The orbital angular momentum M can be either integer or half-integer M=0, ±1/2, ±1, ±3/2, …, which is possible in two dimensions. We have limited ourselves to a detailed discussion of states with J=0, i.e., with the half-integer orbital angular momentum M=−1/2, which is the first to descend to the top of the valence band in the presence of a supercharged impurity in graphene with a gap.

The dependences of the critical charges $Z_{\mathrm{cr}}$ on the homogeneous deformations of graphene on the indicated substrates as well as on the radius of the cutoff of the Coulomb potential at small distances, required for such values of the impurity charge, were calculated. The phases of the scattering of holes by supercritical $Z>Z_{\mathrm{cr}}$ impurities were also calculated, and their behavior under the uniform stretching and compression was studied. The same applies to the poles of the elastic scattering matrix, which determine both the position and the width of the quasi-discrete levels.

In addition, the resonant scattering of the holes by the Coulomb potential modified at small distances in states with J=0 was considered in detail for $Z-Z_{\mathrm{cr}}\ll 1$, i.e., near the top of the valence band. Such scattering is completely analogous to the resonant scattering in the nonrelativistic single-channel problem on the potential with a low-permeable barrier. In both cases, the partial scattering cross-section has a resonant (Breit–Winger) shape, and there are no other inelastic resonance channels. This is an additional proof that the resonances in scattering at $Z>Z_{\mathrm{cr}}$ cannot serve as evidence in favor of the spontaneous production of particle-antiparticle pairs. Thus, the one-particle description is valid not only for $Z<Z_{\mathrm{cr}}$, but also in the supercritical region $Z>Z_{\mathrm{cr}}$. Therefore, it is possible to compare theoretical calculations with experimental data on the current-voltage characteristics obtained by the scanning tunneling microscopy.

Based on such experimental data, it would be possible to conclude whether half-integer orbital angular momenta are realized in the two-dimensional considered systems. In addition, the effective two-dimensional radial Dirac equation with the integer total angular momenta J= ±1, ±2, …, i.e., with half-integer orbital angular momenta M=1/2, ±3/2, …, is exactly the same as the three-dimensional one with the Dirac quantum number ϰ=−J= ∓1, ∓2, …. Thus, one could experimentally establish whether spontaneous production of e^+^e^–^-pairs exist in the supercritical Coulomb problem. However, calculation of the effective dielectric constant of the considered systems is necessary for the direct comparison of experimental data on the spectra of current-voltage characteristics with the theoretical predictions.

## Figures and Tables

**Table 1:** Values of the critical charge $Z_{\mathrm{cr}}(n)$ versus the mechanical tension σ for graphene on the *h*BN substrate. The data is given for the first four levels n=0, 1, 2, 3 with the total angular momentum J=0, and for two kinds of cutoff function corresponding to the values $f_{0}=2$ and $f_{0}=1$, with a cutoff radius $r_{0}=Rl_{D}=15\;Å$.

	$f_{0}=2$			$f_{0}=1$		
σ, %	10	0	−10	10	0	−10
$R=r_{0}/l_{D}$	0.061	0.030	0.012	0.061	0.030	0.012
$Z_{\mathrm{cr}}(0)$	0.115	0.122	0.125	0.155	0.156	0.153
$Z_{\mathrm{cr}}(1)$	0.349	0.378	0.394	0.442	0.466	0.472
$Z_{\mathrm{cr}}(2)$	0.553	0.606	0.640	0.685	0.734	0.756
$Z_{\mathrm{cr}}(3)$	0.743	0.820	0.873	0.909	0.984	1.023
